# A Nested Association Mapping Panel in *Arabidopsis thaliana* for Mapping and Characterizing Genetic Architecture

**DOI:** 10.1534/g3.120.401239

**Published:** 2020-08-11

**Authors:** Marcus T. Brock, Matthew J. Rubin, Dean DellaPenna, Cynthia Weinig

**Affiliations:** * Department of Botany, University of Wyoming, Laramie, WY 82071; † Program in Ecology, University of Wyoming, Laramie, WY 82071; ‡ Donald Danforth Plant Science Center, St. Louis, MO 63132; § Department of Biochemistry and Molecular Biology, Michigan State University, East Lansing, MI 48824; ** Department of Molecular Biology, University of Wyoming, Laramie, WY 82071

**Keywords:** *Arabidopsis thaliana*, GWAS, NAM population, recombinant inbred lines, Multiparent Advanced Generation Inter-Cross (MAGIC), multiparental populations, MPP

## Abstract

Linkage and association mapping populations are crucial public resources that facilitate the characterization of trait genetic architecture in natural and agricultural systems. We define a large nested association mapping panel (NAM) from 14 publicly available recombinant inbred line populations (RILs) of *Arabidopsis thaliana*, which share a common recurrent parent (Col-0). Using a genotype-by-sequencing approach (GBS), we identified single nucleotide polymorphisms (SNPs; range 563-1525 per population) and subsequently built updated linkage maps in each of the 14 RIL sets. Simulations in individual RIL populations indicate that our GBS markers have improved power to detect small effect QTL and enhanced resolution of QTL support intervals in comparison to original linkage maps. Using these robust linkage maps, we imputed a common set of publicly available parental SNPs into each RIL linkage map, generating overlapping markers across all populations. Though ultimately depending on allele frequencies at causal loci, simulations of the NAM panel suggest that surveying between 4 to 7 of the 14 RIL populations provides high resolution of the genetic architecture of complex traits, relative to a single mapping population.

Most phenotypes segregating in natural and agricultural populations are quantitative traits which are commonly regulated by numerous genes of small to moderate effect (Falconer and Mackay 1996; Mackay *et al.* 2009). Dissecting the genetic architecture of quantitative trait variation is fundamental in characterizing evolutionary processes in natural plant populations and is crucial to the development of new agricultural accessions (Bergelson and Roux 2010; Alonso-Blanco *et al.* 2009; Huang and Han 2014). Linkage disequilibrium mapping in segregating progenies (*e.g.*, F_2_, backcross, recombinant inbred lines (RILs), etc.) is a powerful statistical approach used to uncover trait genetic architecture in which marker defined chromosome intervals in an experimental population are tested for statistical associations with phenotypes of interest (Bergelson and Roux 2010; Mackay *et al.* 2009). This traditional mapping strategy has been crucial in furthering our understanding of the complex inheritance of life history traits (*e.g.*, flowering time; Buckler *et al.* 2009; Dittmar *et al.* 2014), physiological traits (*e.g.*, carbon assimilation or water use efficiency; Edwards *et al.* 2011; Hausmann *et al.* 2005) and plant fitness/yield (Malmberg *et al.* 2005). RIL populations (and more recent derivations, *e.g.*, advanced intercrossed RILs, (AIRILs); Balasubramanian *et al.* 2009; Darvasi and Soller 1995) are the product of crossing two parents, followed by numerous generations of selfing and single-seed decent—producing homozygous lines that are a mosaic of chromosomal regions from the parental founders. Once established and genotyped, RIL populations are a convenient community resource with high power to detect segregating QTL at moderate genomic resolution. Additionally, their “immortal” status allows researchers to replicate genotypes within an experiment, improving genotypic trait estimation, and to raise RIL populations across environments, allowing for the examination of genetic variation in trait responsiveness to the environment (*i.e.*, G × E; El-Soda *et al.* 2015).

In spite of the many benefits of RIL populations, they are, by definition, limited to a maximum of two alleles at any one locus, and thus discount a majority of the additive and epistatic interactions that contribute to phenotypic variation in natural populations (Kalisz and Kramer 2008; Rakshit *et al.* 2012). Additionally, the extent of recombination in RILs is largely restricted to early generations as opportunities for detectable recombination events decline as homozygosity via inbreeding increases, limiting the mapping resolution of QTL in these populations. Nested association mapping (NAM) designs seek to mitigate these weaknesses by incorporating numerous RIL populations that share a common recurrent parent (Buckler *et al.* 2009; Yu *et al.* 2008). Specifically, NAM populations allow for a more complete picture of trait genetic architecture via increased sampling of segregating genetic variation, which increases with the number of founding parental lines. Moreover, this strategy benefits from enhanced mapping resolution due to the coupling of historic recombination unique to each founding accession with contemporary recombination during RIL population development. Recent NAM populations have been formed for important agricultural crops (*e.g.*, maize, soybean, rice, etc.), and have been integral to the genetic characterization of important agricultural traits (*e.g.*, flowering time) and fundamental evolutionary mechanisms (*e.g.*, heterosis; Buckler *et al.* 2009; Song *et al.* 2017; Fragoso *et al.* 2017).

*Arabidopsis thaliana* RIL populations have long been used to examine the inheritance of complex traits (Lister and Dean 1993; Reiter *et al.* 1992). This system has many well-known advantages for experimental and natural studies including: short generation time, numerous genomic resources useful in exploring causal genes underlying QTL (*e.g.*, publicly available mutants, resequencing data etc.), numerous segregating populations founded by a broad array of accessions, and translational relevance to agro-ecologically important members of the Brassicaceae family (*e.g.*, *Brassica napus*, *Camelina sativa*, and *Boechera stricta*). The NAM design has been implemented in *A. thaliana* using three RIL populations (Li *et al.* 2011), but would benefit from a larger number of founders as well as a higher density of molecular markers. We expand on the *Arabidopsis* NAM design by utilizing 14 publicly available RIL populations that result from crosses between the recurrent Columbia accession (Col-0) and fourteen alternate founders (Table 1; Simon *et al.* 2008; available at http://publiclines.versailles.inra.fr). We utilize a genotyping-by-sequencing (GBS) approach to augment existing marker genotyping and linkage maps in each RIL population, improving linkage mapping resolution for individual populations. Additionally, we impute an overlapping set of single nucleotide polymorphisms (SNPs) from all parental accessions (Atwell *et al.* 2010), producing a high-density SNP dataset and a joint-linkage map for genome-wide association studies (GWAS) across all 14 populations.

**Table 1:** Metadata and metrics for each of 14 *Arabidopsis thaliana* recombinant inbred line populations (RIL) that share a common recurrent parent (Col-0) and form a nested association mapping population described herein. RIL populations are available at Institut National de la Recherche Agronomique (INRA; Versailles, France; http://publiclines.versailles.inra.fr)

INRA population	Alternate parent	Population size	Total Markers	GBS Markers	Overall Length (cM)	Avg. interval (cM)	Max interval (cM)	Avg. missing data GBS	Avg. missing data post-imputation
2RV	bla1	141	641	563	443.1	0.7	13.9	27.1%	3.2%
3RV	tsu0	145	1254	1175	449.8	0.4	6.4	23.6%	1.4%
4RV	nok1	144	1243	1158	462.6	0.4	7.1	23.0%	1.1%
6RV	ri0	144	1503	1419	489.5	0.3	7.5	16.6%	0.8%
8RV	cvi0	147	1615	1525	634.5	0.4	11.7	22.7%	1.0%
13RV	sha	148	796	711	417.4	0.5	7.9	20.2%	1.3%
17RV	ge0	149	1201	1119	485	0.4	5	23.6%	1.2%
19RV	can0	148	1561	1477	481.5	0.3	6.1	26.0%	1.2%
20RV	bur0	142	1344	1257	433	0.3	5.5	21.5%	1.1%
21RV	blh1	137	1074	1000	485.1	0.5	6.8	23.3%	1.3%
23RV	yo0	150	1351	1269	477.5	0.4	7.2	20.6%	0.8%
27RV	oy0	149	1349	1264	488.7	0.4	9.7	19.7%	1.1%
28RV	jea	147	1435	1348	482.2	0.3	6.5	17.8%	0.8%
29RV	ita0	137	1183	1096	430.8	0.4	9	16.4%	0.9%

## Materials And Methods

### SNP discovery and curation

We selected 14 *Arabidopsis thaliana* RIL populations from the Institut National de la Recherche Agronomique (INRA; Versailles, France) that utilize Col-0 as a common recurrent parent (Table 1). From each population, the most informative 150 RILs were selected, favoring lines with greater number of recombination events and lower levels of heterozygosity (Simon *et al.* 2008); ultimately yielding 2100 unique F_8_ RIL lines. To build high density linkage maps across all 14 *A. thaliana* RIL populations, we took a genotyping-by-sequencing (GBS) approach to SNP discovery. We obtained DNA samples from Versailles, and used GBS methodologies slightly modified from those detailed in Parchman *et al.* (2012). Briefly, we digested each DNA sample with the restriction endonucleases *EcoRI* and *HindIII* and then ligated customized adapters to each fragment containing the Illumina adaptor sequences and 8-10 bp barcode sequences. We substituted *HindIII* (with a 6-base recognition sequence; Table S1) in combination with *EcoRI* for the commonly used *MseI* 4-base cutter (Parchman *et al.* 2012) in order to reduce representation of chloroplast relative to nuclear derived reads as determined *in silico* using the *A. thaliana* reference genome. Ligated fragments were PCR amplified using two separate reactions and resulting products were pooled to limit stochastic effects on relative abundance of fragments. PCR products were then pooled across individuals and libraries were size selected for fragments between 250-700bp using a BluePippin (Beverly, MA, USA). Initial GBS libraries were sent to the RTSF Genomics Core (Michigan State University, East Lansing, MI, USA) and follow-up runs were sent to the Genomic Sequencing and Analysis Facility (University of Texas, Austin, TX, USA). At both facilities, libraries were sequenced on the Illumina HiSeq 2500 platform (1 × 100 bp) and over 1 billion reads were assigned to barcoded samples.

Reads were mapped onto the *A. thaliana* reference genome (TAIR10) using two separate approaches and resulting SNPs calls were merged. First, we used SOAP (SOAPaligner ver. 2.21 and SOAPsnp ver.1.03) in order to set priors on genotype calls based on the probability of expected homozygosity in an F8 RIL population. Second, we utilized BWA’s *aln* and *samse* algorithms (ver. 0.7) to map reads to the TAIR10 reference. We then called SNPs using SAMtools *mpileup* and BCFtools *view* (ver. 0.1.19) algorithms. In both approaches, we retained only uniquely mapping reads and only SNP genotype calls with a read depth of eight or more. We used custom perl scripts to combine SNP calling approaches, merging the novel SNPs from BWA/SAMtools into the SOAPsnp results. Finally, we merged SNPs originally genotyped from each RIL population (INRA; Versailles, France) into our GBS approach after converting INRA SNPs to the TAIR10 coordinate system. We visually inspected each RIL population to confirm that Col-0 *vs.* alternate parental marker states were congruent between our GBS data and markers available at INRA; inconsistent lines were dropped. Additionally, lines with excessive heterozygosity or limited genotyping were dropped; in total 4.1% of lines were excluded bringing the final population to 2028 RILs (Table 1).

### RIL linkage map construction and SNP imputation

For each population, we combined our new GBS SNP markers with existing INRA markers and imported these data into the R/qtl package (Broman *et al.* 2003) with SNP order based on physical location. In each RIL population, we estimated marker map locations (*est.map*; R/qtl) for each chromosome using a Kosambi mapping function (Kosambi 1943). We then imputed missing data across markers in each RIL set using R/qtl’s *fill.geno* function to “fill in” missing genotypes between markers with identical genotypes (ignoring chromosome ends and recombination breakpoint regions). We then removed any imputed genotypes where multipoint marker data estimated genotype probabilities (*calc.genoprob*; R/qtl) were less than 99%.

### QTL simulations in individual RIL populations

To explore how our GBS SNPs improve mapping resolution and efficiency in each RIL population, we ran 1000 simulations, in which small and large effect QTL (∼10% and 30% percent variance explained (PVE), respectively) were simulated at a randomly selected SNP in each RIL population following imputation. In each simulation run, we used the maximum likelihood algorithm in R/qtl’s *scanone* procedure to identify each QTL (testing genome-wide significance following 1000 permutations) and recorded effect size and 1.5 LOD intervals of each QTL. We then dropped the new GBS markers from the dataset and repeated mapping of the simulated trait with the mapping procedure outlined above using only the original INRA markers. We recorded if the simulated QTL was detected in each population using just the INRA markers and, if so, the effect size and 1.5 LOD interval size.

### NAM population SNP imputation and joint-linkage map construction

Because our GBS markers rarely overlapped across populations, we used the robust linkage maps of each individual population to impute a common set of SNP markers across all 14 RIL sets. We utilized the publicly available 250K SNP Arabidopsis dataset for imputation (Horton *et al.* 2012; Atwell *et al.* 2010) because it contained 211,786 overlapping SNPs from 13 of the 14 alternate parents. For the remaining parent, Ita-0, we interrogated publicly available bam files (Durvasula *et al.* 2017) at each SNP location in the 250K dataset to determine Ita-0 marker states.

In each RIL population, we interpolated map positions of the 250K SNPs and again used *fill.geno* to impute the 250K marker states from each parent between GBS markers with identical marker states, *e.g.*, we filled alternate parent SNP states from the 250K dataset into intervals anchored at both ends by alternate GBS marker states. Given that the intervals in the GBS derived linkage maps are on average 0.4cM (see results), this fill in approach has an average imputation error rate of 0.0016% (*i.e.*, the probability of a double crossover in intervals anchored by like parental marker states) and a maximal error rate of 1.93% (in the largest interval across all populations, 13.9cM). All 14 RIL populations were merged together based on imputed, overlapping SNPs and neighboring markers in perfect linkage with respect to both marker state and missing data were reduced to a single entry. These SNPs could be used to map trait genetic architecture via GWA style analyses that control for population structure (Zhou and Stephens 2014; Yu *et al.* 2008). Alternatively, the genetic architecture of complex traits in NAM populations can be resolved via extensions of traditional linkage mapping approaches in concert with a joint-linkage map (Li *et al.* 2011).

Using the final imputed SNP files of our merged NAM population, we also generated a joint-linkage map for all 14 populations. Because recombination events can only be detected between polymorphic SNPs, we selected SNPs for which at least 11 of the 14 alternate parents shared a SNP state and differed from the recurrent parent. SNPs in populations that were not polymorphic for a specific marker were encoded as missing data (Li *et al.* 2011). Markers were imported into R/qtl with ordering based on physical location and genetic map locations were estimated using the Kosambi mapping function (*est.map*; R/qtl).

We estimated patterns of intra- and inter-chromosomal linkage disequilibrium as measured by correlations (r2; TASSEL; Bradbury *et al.* 2007) between 10000 randomly selected imputed SNPs in the 14 RIL populations of the NAM population. To explore how LD changes with the number of RIL populations included in NAM, we dropped seven populations and re-estimated LD values and finally estimated LD within each of these seven populations as a baseline range.

### NAM population power analyses

The ability to identify and characterize segregating genetic variation in NAM analyses depends upon the QTL effect size, the degree of linkage between causal gene and adjacent markers, the frequency of each allele in the founding parental lines (and subsequently in the RIL progeny of that specific cross), and the overall sample size of RILs in each allelic class from which to estimate mean and standard error. Moreover, the cost-benefit relationship of phenotyping multiple RIL populations will vary given both the trait (*e.g.*, how difficult the trait is to measure) and its genetic architecture. To broadly examine the ability of different combinations of the 14 Arabidopsis RIL populations in resolving QTL across a range of effect sizes, we simulated two QTL scenarios. First, we selected all markers where all alternate parental lines were identical and differed from the recurrent Columbia parental line (*i.e.*, all RIL populations segregated at these SNPs). We randomly selected a SNP marker and simulated a QTL at this marker for all RILs in all 14 populations; we then created 13 additional datasets of this trait by progressively dropping a randomly selected RIL population. This process was repeated 1000 times for each of seven QTL effect sizes (5%, 7.5%, 10%, 15%, 20%, 40%, 80% PVE). Second, we selected markers that varied in only one of the 14 RIL populations to explore how power to detect a rare causal QTL in a single population was influenced by adding additional invariant populations (which would influence sample size and trait mean estimation). We followed a similar approach to that above by simulating QTL of seven effect sizes for a randomly selected marker and repeating this 1000 times for each PVE category. In this second simulation, PVE settings were consistent with those above; however, because only one population varied, target PVEs were only realized in the single segregating population. We again created 14 separate datasets by progressively dropping a randomly selected RIL population that was not polymorphic.

For each simulation scenario, we used a univariate linear mixed model to perform a likelihood ratio test while controlling for relatedness within and across RIL populations for each of the 1000 simulated traits for each of the 14 datasets. We recorded if a significant effect was detected for the SNP selected during QTL simulation (GEMMA ver. 0.97; Zhou and Stephens 2014), using a LOD score of ∼3 (P-value = 0.0001; Nyholt 2000) as a generally accepted, albeit somewhat liberal, genome-wide significance threshold.

### Data availability

In addition to data within this manuscript, we present supplemental files via FigShare. Fig. S1 and Fig. S2 contain linkage map and LD plots of RILs and the NAM population, respectively. Table S1 contains sequences of custom adapters described in the methods. We have submitted all individual RIL linkage maps, imputed SNPs across all 14 NAM populations, and a joint-linkage map where at least 11 alternative parents share a common SNP state to the dryad repository (https://doi.org/10.5061/dryad.c2fqz614w). Supplemental material available at figshare: https://doi.org/10.25387/g3.12683750.

## Results & Discussion

Most traits segregating in natural and agricultural populations have a quantitative genetic basis, which have often been characterized through the use of traditional LD mapping (Doerge 2002; Mackay 2001) and more recent association mapping approaches (Yu *et al.* 2008). RIL populations have long been used to detect QTL of broad chromosomal regions with strong statistical power, which results from loci segregating at intermediate allele frequencies and the ability to robustly estimate genotypic means through averaging replicate plants of the same multilocus genotype (Bergelson and Roux 2010). However, a single RIL population fails to characterize much of the genetic diversity segregating across natural populations because each RIL population can carry at most two alleles at any causal locus and will be fixed at the majority of causal loci segregating in the species. Nested-association mapping panels (NAM) consist of multiple RIL populations that all share a common, recurrent parental line; this design should enhance segregating genetic diversity, power, and mapping resolution while maintaining the utility and immortality of RIL populations (Yu *et al.* 2008). Here we present GBS genotyping of 14 *Arabidopsis* RIL populations that all share the well-characterized recurrent parent Col-0. These GBS data were used to estimate robust linkage maps in each population and subsequently leveraged to impute shared whole-genome SNP data into each RIL set, creating a large 14-population NAM panel of *Arabidopsis*.

### RIL Linkage map construction, SNP imputation, and QTL simulations

**Figure 1:**
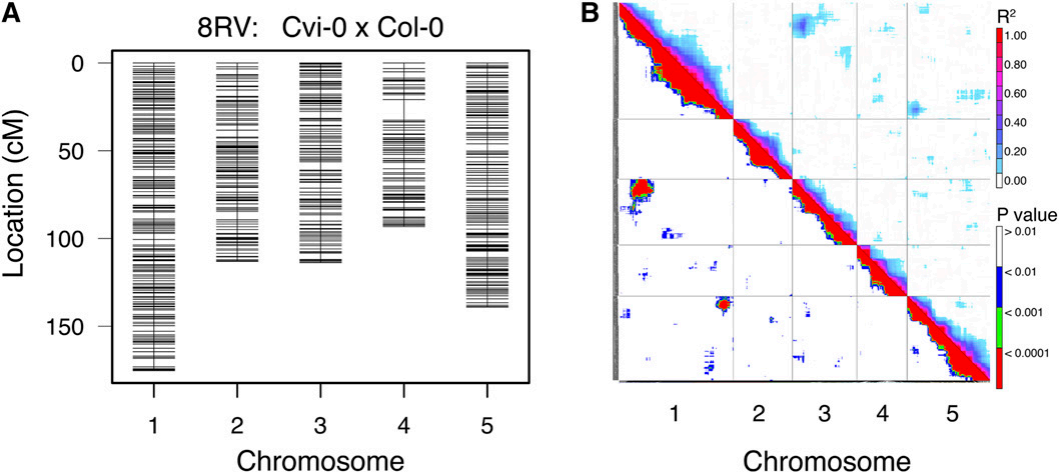
A) Linkage map for the Cvi-0 x Col-0 *Arabidopsis thaliana* RIL population (8RV: http://publiclines.versailles.inra.fr/rils/index) using 1615 total SNPs (both original INRA and new GBS identified SNPs) following conservative imputation. B) Linkage disequilibrium plot of intra- and inter-chromosomal LD in Cvi-0 x Col-0 as measured by SNP-SNP Pearson product moment correlation coefficients (*r*^2^; top right of diagonal) with associated significance tests (p-value; lower left of diagonal). See Fig. S1 for linkage maps and LD plots of remaining 13 *Arabidopsis* RIL populations referenced in Table 1.

We identified an average of 1170 SNPs per RIL population (range: 563-1525 SNPs). Combining our new GBS markers with the original markers developed at INRA (range: 74-90 markers), we estimated new linkage maps in each of the 14 populations resulting in an average marker spacing of 0.4 cM (range: 0.3-0.7 cM) and an average maximum spacing of 7.9 cM (range: 5.0-13.9 cM; Table 1; individual RIL linkage maps available at Dryad: Brock *et al.* 2020). These results substantially add to previous linkage map versions (available at http://publiclines.versailles.inra.fr; Simon *et al.* 2008), reducing the average and maximum interval size by 92.3% and 40.7%, respectively. Our improved RIL linkage maps are comparable to other recently constructed maps that also used a GBS approach, producing high-density genetic maps with increased genomic resolution of recombination events (*e.g.*, Serin *et al.* 2017). Rates of missing genotype data at our GBS SNPs varied across RIL populations, averaging 21.6% (range: 16.4–27.1%; Table 1); however, following a conservative “fill in” imputation approach, which ignored breakpoints and chromosome ends, levels of missing data fell to an average of 1.2% across all RIL populations (range: 0.8–3.2%). Recombination fraction plots (not presented) support using physical location from the Col-0 TAIR10 reference genome to order markers in each population. Moreover, we note regions with significant inter-chromosomal linkage disequilibrium in 8 of the 14 RIL populations (Table 2; Figure 1; Fig. S1). Several of these instances of inter-chromosomal LD are pronounced—lacking one of the four possible allelic combinations for these interacting regions.

**Table 2:** Regions of significant linkage disequilibrium and specific allelic combinations with distorted low frequency (REF and ALT indicate Col-0 and alternate parent alleles, respectively) in each of eight *Arabidopsis thaliana* recombinant inbred populations. Regions are denoted as chromosome @ nucleotide position (in MB)

INRA population	Alternate parent	LD region 1	low frequency allelic combination 1	LD region 2	low frequency allelic combination 2
3RV	tsu0	3@17.6MB x 4@ 1.2MB	13.3% REF & REF		
4RV	nok1	1@13.6MB x 5@ 8.5MB	0% REF & ALT		
6RV	ri0	1@ 2.2MB x 3@23.1MB	11.1% REF & REF		
8RV	cvi0	1@ 6.6MB x 3@ 2.2MB	2.3% REF & ALT	1@27.1MB x 5@ 3.4MB	0% REF & ALT
13RV	sha	4@13.2MB x 5@26.7MB	3.7% REF & ALT		
21RV	blh1	4@ 0.1MB x 5@ 4.4MB	8.5% REF & REF		
28RV	jea	4@10.9MB x 5@ 4.0MB	2.1% REF & ALT	2@14.9MB x 4@12.2MB	16.1% REF & ALT
29RV	ita0	1@27.1MB x 5@ 3.9MB	1.6% REF & ALT	4@ 1.4MB x 5@ 3.1MB	3.4% ALT & ALT

Segregation distortion has been previously reported in many of these RIL populations (Simon *et al.* 2008), and several have subsequently been well-characterized as prominent examples of Dobzhansky-Muller incompatibilities. Genetic incompatibilities between Chr 1 and Chr 5 identified in Cvi-0 x Col-0 and Ita-0 x Col-0 have been shown to result from reciprocal loss of a historically duplicated essential gene. Specifically, the combination of a homozygous null copy of the histidinol-phosphate aminotransferase 1 gene (*HPA1*) on Chr 1 (donated by Col-0) combined with a homozygous null copy of *HPA2* on Chr 5 (donated by Ita-0 or Cvi-0) results in recessive embryo lethality and lines with these allelic combinations were lost during RIL development (Bikard *et al.* 2009; Simon *et al.* 2008). Similar genetic mechanisms have been shown to produce incompatibilities in Sha x Col-0 between null and epigenetically silenced versions of paralogous duplications of a folate transporter gene (*FOLT*) present on Chr 4 and Chr 5 (Durand *et al.* 2012) as well as in Nok-1 x Col-0 RILs that exhibit incompatibilities between variation in the presence of functional alleles and silenced epialleles of the tRNA-specific adenosine deaminase 3 gene (*TAD3)* found on Chr 5 and Chr 1 (Agorio *et al.* 2017; Balasubramanian *et al.* 2009). Additional extreme distortions in Jea x Col-0 and in Ita-0 x Col-0 (in both cases between regions on Chr 4 and Chr 5) suggest additional incompatibilities that are, as yet, unexplored.

In simulations of small and large effect QTL (average PVE: 10.2% and 28.8%, respectively) within each individual RIL family, the addition of our GBS markers reduced 1.5 LOD intervals by an average of 23.3% (range: 13.4–34.4%) and 56.6% (range: 40.4–63.3%), respectively (Table 3). These small and large effect QTL average genetic intervals translate to 5.1 to 7.6 Mb and 1.5 to 2.9 Mb, respectively, for our estimated 1.5 LOD support intervals in which we have ∼95% confidence of localizing the simulated QTL (Alonso-Blanco *et al.* 1998; Dupuis and Siegmund 1999). Although the additional GBS markers did not appreciably improve power to detect large-effect QTL (Table 3), the GBS datasets were on average 31.2% more likely to detect a simulated small-effect QTL relative to analyses with only the original INRA markers. QTL mapping resolution is correlated with the number of recombination events in a mapping population, which has led to alternative mapping population designs with additional generations of intercrossing (*e.g.*, advanced intercross RILs (AI-RILs); Balasubramanian *et al.* 2009; Darvasi and Soller 1995). Additional genotyping within the individual RIL populations is unlikely to dramatically improve resolution beyond the gains presented here; instead, future efforts should incorporate additional lines (*i.e.*, beyond the ∼150 per population here) to capture additional recombination events while concomitantly improving power through more accurate estimation of genotype means.

**Table 3:** Results of simulating small and large effect QTL in each of 14 *Arabidopsis thaliana* recombinant inbred populations illustrating increased power of detection and enhanced interval resolution of linkage maps with GBS SNPs in comparison to original INRA maps

Small Effect QTL							
INRA population	Alternate parent	Mean QTL PVE		Mean 1.5 LOD Interval (cM)		Perc. Reduction of QTL Interval	Perc. QTL detected by INRA
		GBS	INRA	GBS	INRA		
2RV	bla1	10	9.7	25.2	29.1	−13.4	74.2
3RV	tsu0	10.1	9.6	23.3	28.1	−17.1	75.7
4RV	nok1	10.5	9.9	21.3	28.5	−25.3	75.9
6RV	ri0	10.3	9.5	24.5	34.1	−28.2	79.1
8RV	cvi0	10.6	9.8	24.2	36.9	−34.4	78
13RV	sha	9.8	9.3	21	27.1	−22.5	80.3
17RV	ge0	9.9	9.2	21.9	28.4	−22.9	78.2
19RV	can0	10.1	9.5	22.6	30.6	−26.1	75.7
20RV	bur0	10.1	9.5	23.8	32.8	−27.4	75.5
21RV	blh1	10.7	10	24.7	31.6	−21.8	68.7
23RV	yo0	10.2	9.4	21.1	29.9	−29.4	74.4
27RV	oy0	10	9.4	24.5	29.2	−16.1	74.9
28RV	jea	10.1	9.4	22.3	28.6	−22	78.6
29RV	ita0	10.7	10	22	27.5	−20	76.9

Large Effect QTL							
INRA population	Alternate parent	Mean QTL PVE		Mean 1.5 LOD Interval (cM)		Perc. Reduction of QTL Interval	Perc. Of QTL detected by INRA
		GBS	INRA	GBS	INRA		
2RV	bla1	28.9	26.6	9.7	16.3	−40.4	98
3RV	tsu0	29.2	26.3	7.2	15.9	−55.1	99.3
4RV	nok1	28.4	25.5	7.1	16.1	−55.5	98.4
6RV	ri0	29	25.9	6.7	16.8	−60.3	98.5
8RV	cvi0	28.7	25	7.1	19.5	−63.3	97.8
13RV	sha	28.5	25.9	7.6	15.7	−51.7	98.8
17RV	ge0	28.9	25.6	6.9	17.1	−59.8	98.9
19RV	can0	28.9	25.8	6.7	17.6	−62.2	98.5
20RV	bur0	28.7	26	6.8	15.1	−54.8	98.2
21RV	blh1	29.1	25.9	7.8	18.3	−57.6	98.6
23RV	yo0	29.1	25.8	6.8	17.6	−61.3	99.2
27RV	oy0	29.1	26	7	16.5	−57.7	97.7
28RV	jea	28.9	25.7	6.6	16.6	−60	98.9
29RV	ita0	28.2	25.3	8	16.8	−52.4	98

### NAM population SNP imputation, joint-linkage map construction, and power analyses

We imputed SNPs from the 250K *Arabidopsis* dataset (Horton *et al.* 2012; Atwell *et al.* 2010) into our robust linkage maps of each RIL population, resulting in an imputed NAM dataset of 181,579 SNPs across all five chromosomes for the 2028 RILs (NAM imputed SNPs available at Dryad: Brock *et al.* 2020). Following imputation only 5.6% of the NAM SNPs were missing and given our imputation approach most missing data are found at the chromosome ends where anchoring GBS markers were found in progressively fewer populations. In addition to GWA analyses that test for phenotype associations across a large imputed SNP dataset (*e.g.*, GEMMA; Zhou and Stephens 2014), other approaches use a joint-linkage map to identify QTL residing in marker-defined intervals. Using SNP markers where at least 11 of the 14 alternate parents differed from the marker state of the recurrent Col-0 parent, we built a joint-linkage map, which had an average interval size of 0.1cM with a maximum interval size of 2.9cM (Fig. S2; NAM joint-linkage map available at Dryad: Brock *et al.* 2020).

**Figure 2:**
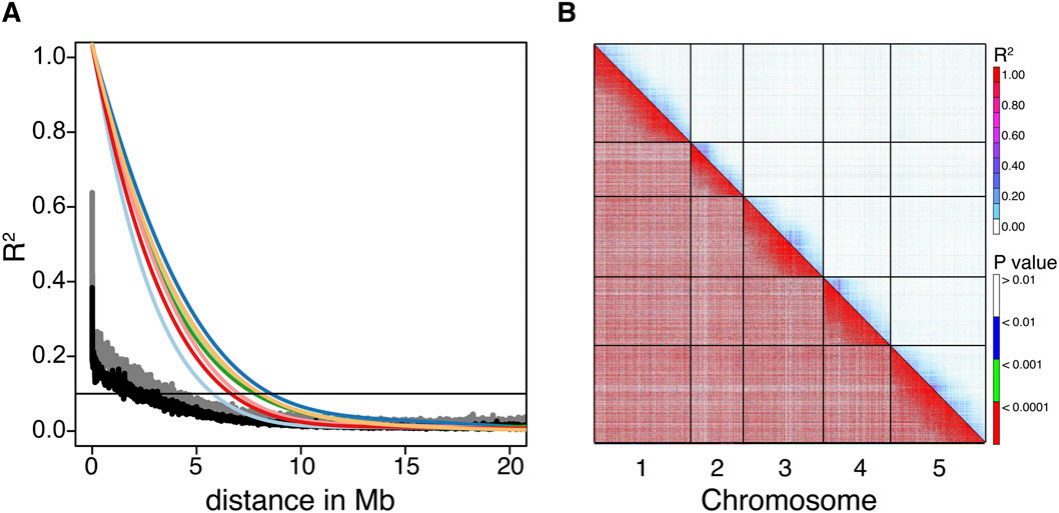
A) Linkage disequilibrium (LD; *r*^2^) as a function of distance across all five *Arabidopsis thaliana* chromosomes of combined and separate RIL populations. Black lines illustrate LD in all 14 RIL populations of the NAM panel described herein; gray lines illustrate LD in a subset of just 7 RIL populations, and colored best fit lines illustrate the range of LD in each of the 7 populations. B) LD plots illustrating inter-chromosomal associations that are typically weak (*r*^2^; top right of diagonal) but commonly significant (p-values; lower left of diagonal).

Within individual RIL populations there was, not surprisingly, extensive intra-chromosomal LD, falling to an *r*^2^ value of 0.1 between distances of ∼5.8 - ∼8.6Mb in a sample of 7 of the 14 RIL populations (Figure 2A); however, merging multiple RIL populations resulted in intra-chromosomal LD decaying over much shorter distances, falling to an *r*^2^ value of 0.1 at ∼3.5Mb and ∼2.1Mb when combining 7 or all 14 RIL populations, respectively. Inter-chromosomal LD across the imputed SNPs of all 14 RIL populations were commonly significant due to the large number of RILs; however, the strength of the associations was weak (mean *r*^2^ = 0.0098; 95% CI = 0.00983 – 0.00985; Figure 2B).

Weaker LD observed in the combined NAM population relative to individual RIL populations largely arises from the action of historical recombination events and evolutionary forces differentially shaping the genomes of parental lineages, which reduces SNP-SNP correlations as increasing numbers of RIL populations are added. The more rapid decay in intra-chromosomal LD should improve mapping resolution in *Arabidopsis* NAM analyses (Yu *et al.* 2008). LD patterns in this NAM panel are similar to those observed in the *Arabidopsis* Multiparent Advanced Generation Inter-Cross (MAGIC) lines (Scarcelli *et al.* 2007; Kover *et al.* 2009); a RIL population generated by intercrossing 19 *Arabidopsis* parental founders and resulting offspring for four generations followed by six additional generations of inbreeding. In the MAGIC population, *r*^2^ falls to 0.1 around 4.5Mb suggesting that the NAM population may have slightly finer mapping resolution; however, given the additional generations of intercrossing in the MAGIC population, these apparent differences in LD may stem from ascertainment bias of SNPs describing LD in Kover *et al.* (2009) relative to the 10K randomly selected SNPs following imputation used here.

**Figure 3:**
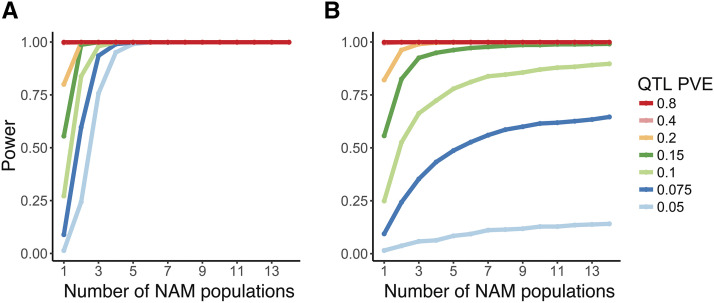
Plots of the ability for increasing numbers of randomly combined *Arabidopsis thaliana* RIL populations to detect simulated QTL of variable effect sizes (see legend for average simulated percent variance explained). Simulations bracket the range of possible allele frequencies found across the 14 RIL populations, testing two scenarios: A) simulated QTL are shared among all alternate parents and so are found to vary in all RIL populations and B) simulated QTL vary within only a single RIL population.

We used 181,579 SNP dataset to explore the ability of different subsets of the NAM population to resolve simulated QTL of variable effect sizes (5–80% PVE; Figure 3). We selected two extreme cases with which to explore NAM power: causal SNPs that are segregating in all RIL populations or just a single population. Our linear mixed model results suggest that power to resolve small to moderate effect QTL increases substantially when comparing single RIL populations to NAM panels consisting of three or more RIL populations (Figure 3A). Strikingly, for causal effect loci that are shared among alternate parents, gains in power diminish beyond NAM panels of four RIL populations; however, given the lack of *a priori* knowledge regarding trait genetic architecture and the fact that inclusion of multiple parents increases the likelihood of capturing a greater allelic diversity of causal loci, NAM panels of seven populations should provide a more robust characterization of trait genetic architecture while limiting costs of space and phenotyping time required for raising all 2028 RILs (Figure 3B).

INRA’s formation and curation of RIL populations that include the recurrent Col-0 parental line coupled with our new GBS SNP data and linkage maps adds to a growing number of segregating progeny. Given available multi-parental *Arabidopsis* populations, researchers may gravitate toward either using the MAGIC lines (Kover *et al.* 2009) or the NAM panel described here; both are powerful populations for exploring the genetic architecture of complex traits and have obvious advantages over more traditional F_2_ and single RIL populations. The MAGIC lines (at 527 MLs as described in Kover *et al.* 2009) should offer a larger sampling of genetic variation given the intercrossing of 19 founding parental lines—an advantage when dissecting trait genetic architecture of moderate to large effect-size QTL. Yet, as minor allele frequency (MAF) declines in the MAGIC (to the theoretical minimum of ∼1/19) the number of MLs that could carry a rare allele also declines (toward ∼28 of 527 MLs). This low replication may impede detection of allelic effects for low to moderate effect-size QTL. The minimum MAF in any subset of the NAM panel yields at least ∼75 RILs with which to estimate allelic effects, and, as such, researchers interested in discovery of low to moderate effect alleles may be better served with the NAM panel.

QTL detection ultimately depends on the presence of genetic variation from founders of a segregating population as well as the underlying trait genetic architecture. Screening parental lines (of NAM and MAGIC populations) can further QTL discovery by ensuring maximal phenotypic variation in traits of interest segregating in a specific mapping population. Researchers utilizing a subset of the full NAM panel could also benefit from selecting RIL sets derived from the most negatively and positively deviating parental lines for a trait of interest. Together with other advanced segregating progeny (Balasubramanian *et al.* 2009; Kover *et al.* 2009), this NAM panel should help advance a research community interested in identification of highly-resolved QTL regulating complex traits as well as the characterization of trait genetic architecture across environments.
